# ASCOT: A web tool for the digital construction of energy minimized Ag, CuO, TiO_2_ spherical nanoparticles and calculation of their atomistic descriptors

**DOI:** 10.1016/j.csbj.2024.03.011

**Published:** 2024-03-12

**Authors:** Panagiotis D. Kolokathis, Evangelos Voyiatzis, Nikolaos K. Sidiropoulos, Andreas Tsoumanis, Georgia Melagraki, Kaido Tämm, Iseult Lynch, Antreas Afantitis

**Affiliations:** a NovaMechanics MIKE, Piraeus 18545, Greece; b NovaMechanics Ltd, Nicosia 1070, Cyprus; c Entelos Institute, Larnaca 6059, Cyprus; d Division of Physical Sciences and Applications, Hellenic Military Academy, Vari 16672, Greece; e Institute of Chemistry, University of Tartu, Tartu 50090, Estonia; f School of Geography, Earth and Environmental Sciences, University of Birmingham, Birmingham B15 2TT, United Kingdom

**Keywords:** Ag, CuO, TiO_2_, Tenorite, Anatase, Rutile, Descriptors, Nanoparticle, Energy Minimization, Automation

## Abstract

ASCOT (an acronym derived from Ag-Silver, Copper Oxide, Titanium Oxide) is a user-friendly web tool for digital construction of electrically neutral, energy-minimized spherical nanoparticles (NPs) of Ag, CuO, and TiO_2_ (both Anatase and Rutile forms) in vacuum, integrated into the Enalos Cloud Platform (https://www.enaloscloud.novamechanics.com/sabydoma/ascot/). ASCOT calculates critical atomistic descriptors such as average potential energy per atom, average coordination number, common neighbour parameter (used for structural classification in simulations of crystalline phases), and hexatic order parameter (which measures how closely the local environment around a particle resembles perfect hexatic symmetry) for both core (over 4 Å from the surface) and shell (within 4 Å of the surface) regions of the NPs. These atomistic descriptors assist in predicting the most stable NP size based on lowest per atom energy and serve as inputs for developing machine learning models to predict the toxicity of these nanomaterials. ASCOT's automated backend requires minimal user input in order to construct the digital NPs: inputs needed are the material type (Ag, CuO, TiO_2_-Anatase, TiO_2_-Rutile), target diameter, a Force-Field from a pre-validated list, and the energy minimization parameters, with the tool providing a set of default values for novice users.

## Graphical Abstract

**Figure:**
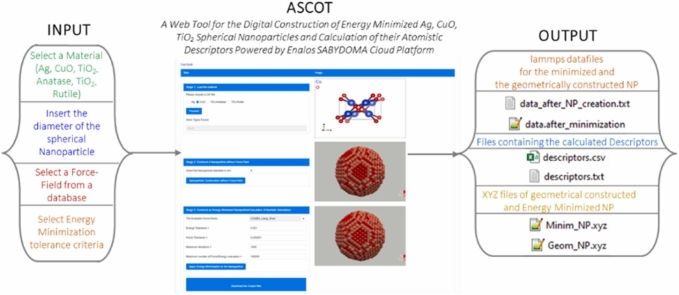

## 1 Introduction

Nanomaterials (NMs) are materials having at least one of their dimensions between 1 and 100 nm, and are considered an enabling technology as they have a wide range of applications across consumer and industrial areas including in medicine, food and energy [1]. Despite the well-documented advantages of NMs over traditional materials [2], [3], comprehensive risk assessments of their potential hazards are often lacking, primarily due to the high cost and complexity of the necessary experiments coupled with the need for revision of the regulatory Test Guidelines to account for the non-equilibrium properties of NMs [4]. This challenge is compounded by the variability of available NMs toxicity data, as the dynamic nature of NMs and their myriad interactions with their surroundings including biomolecule corona formation, mean the same NM can behave differently depending on the dispersion approach and medium composition (see for example Sauer et al. [5], Hadrup et al. [6] Guo et al. [7]).

Recent advances in data-centric and statistical modeling have significantly enhanced the prediction of nanomaterial (NM) toxicity and enabled computational filling of gaps in experimental data [8], [9], [10]. The rapid computational progress underlines the growing need for automated user-friendly computational workflows that can be operated by non-specialists to screen candidate NMs as part of a safe and sustainable by design strategy, and enable regulation to keep pace with technological development, given that NMs are already prevalent in daily applications [11]. Among the most widely used NMs, based on their high production volumes (see e.g., Gottschalk et al. [12]), are Ag, CuO, and TiO_2_ NPs, which are incorporated into nanofluids for high-performance thermal applications such as heating/cooling systems, chemical process heat exchangers, electronics, microchips, and medical applications [13]. These NPs enhance fluid properties by leveraging their remarkable thermal stability and conductivity. ASCOT is particularly focused on these materials, which are also widely used in nanocomposites to improve polymer matrix properties, and which formed a case study in the SABYDOMA project.

ASCOT aims to address the knowledge gap in NMs risk assessment through provision of a platform for computational modeling using fully *in silico* NMs whose properties can be varied systematically, in order to explore the materials parameter space and the effect of different NMs properties on their resulting cellular toxicity. ASCOT not only identifies NMs properties contributing to their toxicity but also provides quantitative data to supplement experimental findings. Factors like NM size, long recognized as crucial, shape and orientation [7] and surface charge (since cationic NMs are known to be more toxic than their anionic counterparts), are considered. Additionally, the presence and symmetry of crystalline phases influences toxicity, with lower toxicity observed in amorphous TiO_2_ NMs and higher levels associated with certain crystalline forms [10].

Among the various modelling approaches for predicting NM toxicity is the quantitative structure-activity or structure-property relationships (QSAR/QPAR) modelling, whereby material’s effects or properties can be predicted from knowledge of their chemical structure. QSAR/QPAR and data-centric models utilizing statistical and machine learning algorithms have shown success in complex environments based on structural and molecular properties, and the established processes for their documentation (via QSAR model report forms), which provide confidence to users based on the model provenance information [14], [15], [16], [17]. These models, however, are often constrained by the size of the available datasets they can be trained and tested on, unlike the more comprehensive and computationally intensive 1st principles methods like density functional theory and atomistic simulations [18]. The need for larger, integrated NMs property and toxicity datasets is evident, and is achievable only through widespread implementation of data management practices that produce FAIR (Findable, Accessible, Interoperable, and Reusable) datasets for further use [19]. Data are a) findable if a unique identifier has been assigned to them, b) accessible if can be reached and are retrievable by their unique identifier, c) interoperable if they can be integrated with other data/workflows and d) reusable if they have a clear license to govern the terms of reuse. Application of automated computational workflows that include data mining and curation steps or data enrichment via generation of computational descriptors, along with generation of toxicity or other end-point predictions, and their upload into FAIR NMs databases is also vital [20]. Thus, the primary goal of ASCOT is the creation of an automated computational workflow for a) the digital construction of spherical Ag, CuO and TiO_2_ NPs and b) the calculation of atomistic descriptors related to the chemical structure of the NPs that can be used as input for QSAR model development.

## 2 Methodology

The workflow presented herein, using ASCOT to digitally construct and analyze energy-minimized Ag, CuO, TiO_2_ (Anatase) and TiO_2_ (Rutile) NPs, uses computational proxies of NMs to explore the interaction space computationally, as a first step towards a fully *in silico* approach to nanosafety assessment and development of safe and sustainable by design (SSbD) NMs. ASCOT integrates the LAMMPS molecular dynamics simulator [21] with the OpenKIM database [22], enabling the efficient computation of NM descriptors, and enables direct upload of the calculated properties for each NM specification into the NanoPharos database [23], streamlining the re-use of these digital NMs and their descriptors in other models and workflows, including QSAR model development.

### 2.1 Selection of Ag, CuO, TiO_2_-Anatase and TiO_2_-Rutile crystallographic information files

To facilitate molecular simulations of Ag, CuO, TiO_2_-Anatase, and TiO_2_-Rutile NPs, their digital construction is a prerequisite. These materials, being crystalline, are characterized by a fundamental unit known as the unit cell which describes the smallest lattice (set of identical points) that can represent an entire crystal. The bulk phase of these materials can be digitally reconstructed by replicating the unit cell along all three Cartesian axes, leading to the formation of their bulk structure. To identify these unit cells, single-crystal and/or powder X-ray/Neutron crystallography is employed to ascertain the types of atoms and their coordinates, which accurately replicate their diffraction patterns. This information is stored in Crystallography Information Files (CIF), which follow specific formatting rules. CIF files contain the atomic coordinates of an asymmetric cell and the symmetry rules needed to construct the unit cell from this asymmetric cell. The asymmetric cell is the essential component required to build a unit cell through the application of specific symmetry rules (i.e., mathematical operations). These digitally reconstructed unit cells for each crystal are archived in the CIF format in crystallographic databases such as the Crystallography Open Database (COD), each with a unique identifier and a common set of metadata that describes the source of the data (provenance) and the parameters of the unit cell [24].

Regarding Ag (silver), the COD houses eighteen CIF files. Sixteen of these correspond to the space group Fm-3 m, which is associated with a face-centered cubic cell. This space group represents the commonly occurring phase of Ag known as native silver [25]. The remaining two files belong to the space group P63/mmc, representing an antimonian (Sb) variety of silver with a hexagonal unit cell. This variety, found in antimony (Sb) and silver (Ag) mixtures, is a rare nanophase [26]. To avoid confusion among non-expert users, ASCOT excludes this variant, focusing instead on the more prevalent native silver structure. For digital construction of Ag NPs, ASCOT utilizes the CIF file with COD ID 1509146, which differs slightly (about 1‰) from the other CIF files. However, this minor difference does not affect the coordinates of the energy-minimized, digitally constructed atoms of the resulting Ag NP in which we are most interested, because according to statistical mechanics [27], the configuration of the NP after energy minimization is the most probable configuration at the specific (fixed) temperature. During the energy minimization process, the NP’s atoms move to new positions so that the NP’s energy is minimized. Energy minimization using any of the native silver CIF files leads to the same energy minimum. The CIF file with COD ID 1509146 represents a low-temperature structure, closely aligning with the energy-minimized state (zero temperature). CIF files of higher temperature structures correspond to the average coordinates of the NPs' atoms. Structures derived from lower temperatures are expected to reach their energy minimum in fewer steps during the energy minimization process, as they start from a position closer to this minimum. If the initial structure is far from the global energy minimum, it may become trapped in local minima during the energy minimization stage. Consequently, the final constructed NP structure may differ significantly from the actual structure. Therefore, selecting the appropriate CIF file is critical for accurate digital construction of the NPs. ASCOT has pre-selected the most suitable structures for use by non-expert users, while detailed information about these files is available in ASCOT’s extensive manual for experienced users.

**Fig. 1:**
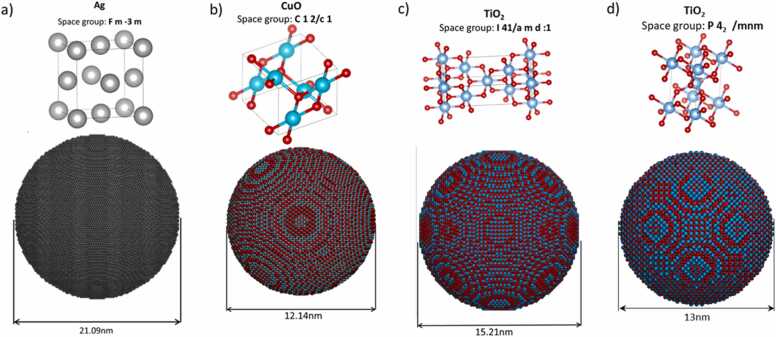
Unit cells of a) Ag (native silver), b) CuO (tenorite), c) TiO_2_ (Anatase) and d) TiO_2 (_Rutile) and spherical NPs built using these unit cells via ASCOT.

For CuO, the COD currently lists sixteen structures under the space group C12/c1 (monoclinic unit cell) and two structures under the space group C1c1 (monoclinic unit cell). The C1c1 space group is derived by modifying some symmetry rules of the C12/c1 group, suggesting a strong relationship between these CIF files, albeit with minor changes in the positions of the CuO unit cell atoms due to this altered symmetry. The CIF file with COD ID 1011148, produced by Tunnel et al. [28], has been selected for use in ASCOT, following a similar rationale as with Ag. The unit cells from these selected CIF files are depicted in Fig. 1, alongside NPs of various diameters constructed via ASCOT.

In the case of TiO_2_, several stable crystalline phases exist, including anatase, rutile, brookite, and akaogite, each corresponding to the same chemical formula but not directly related to each other. According to the pressure-temperature phase diagram by Akrami et al. [29] and Hanaor and Sorrell [30] for TiO_2_ in air, rutile is the most stable form at high temperatures, while anatase prevails at lower temperatures. In contrast, in the absence of air, rutile is more stable than anatase in a vacuum. The coexistence of less stable phases alongside the more stable ones is possible due to energy barriers that impede their transition to a more stable phase. Furthermore, the pH of the surrounding medium also influences the stability of these phases; for instance, reducing the pH increases the proportion of the rutile phase in a TiO_2_ mixture [31]. ASCOT, designed for non-expert users, includes only the rutile and anatase phases, as these are the most likely to occur in real world nanoscale materials according to the aforementioned pressure-temperature phase diagram. TiO_2_-Anatase belongs to the I41/amd space group, with eleven anatase registrations in the crystallography database. Among these, the CIF file with COD ID 1010942, created by Robert Parker [32], has been selected as the default in ASCOT. The TiO_2_-Rutile phase falls under the P42/mnm space group, with eleven rutile registrations in the database. The default CIF file for rutile, COD ID 1532819 by Okrusch et al. [33], has been chosen for use in ASCOT. The occupancy factors for every atom in these CIF files are set to 1, ensuring that no atoms share the same fractional coordinate (occupancy factors less than one) or are superimposed with others (occupancy factors more than one). This precise specification of atom positions allows ASCOT’s algorithm to operate in a strictly deterministic manner, meaning that given a known set of initial conditions, future states can be computed and that there are no random occurrences.

### 2.2 Digital geometrical construction of spherical Ag, CuO and TiO_2_ NPs

**Fig. 2:**
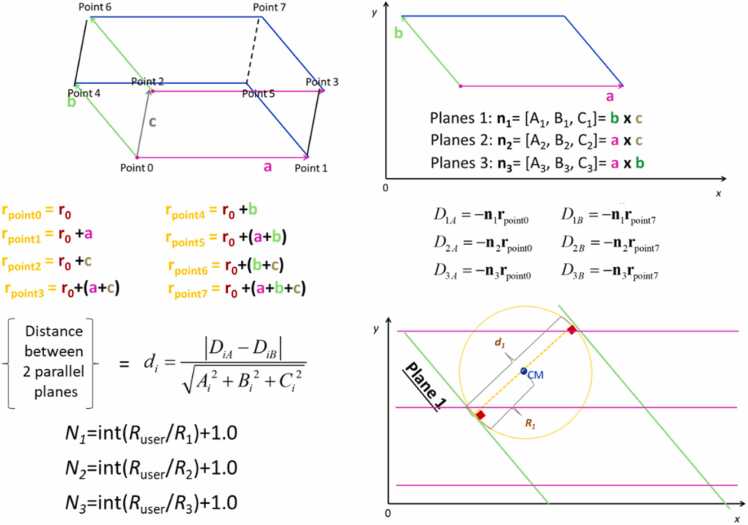
Calculation of the minimum number of unit cells per x, y, z direction (= *N*_1_, *N*_2_, *N*_3_) that are needed to make a spherical NP with radius *R*_user_. The unit cell vectors **a**, **b**, **c** are illustrated with pink, green and brown colours respectively.

In addition to the fractional coordinates and space groups discussed in Section 2.1, each unit cell is defined by its lattice vectors, as illustrated in Fig. 2 (denoted as a, b, and c vectors). Fig. 2 also shows that the normal vectors, perpendicular to the unit cell planes, can be calculated using their cross-product. Moreover, each point within the unit cell is defined by these vectors. The methodology outlined in Fig. 2 enables the determination of the distance between two parallel planes. This measurement is crucial for calculating the minimum number of times a unit cell must be replicated in each (x, y, z) direction to construct a box that can encompass a sphere of a specific diameter. This approach is vital to avoid creating excessively large boxes that would contain atoms with coordinates occupying substantial RAM (random-access memory) space. The process depicted in Fig. 2 is essential for identifying the smallest box size necessary to form a sphere thereby preventing potential memory issues. Furthermore, this approach is critical for maintaining the stability of the ASCOT web tool, particularly when multiple users access and operate it simultaneously. Use of the minimum number of unit cells per x,y,z direction ensures that the tool remains stable and responsive under varying loads and usage scenarios.

**Fig. 3:**
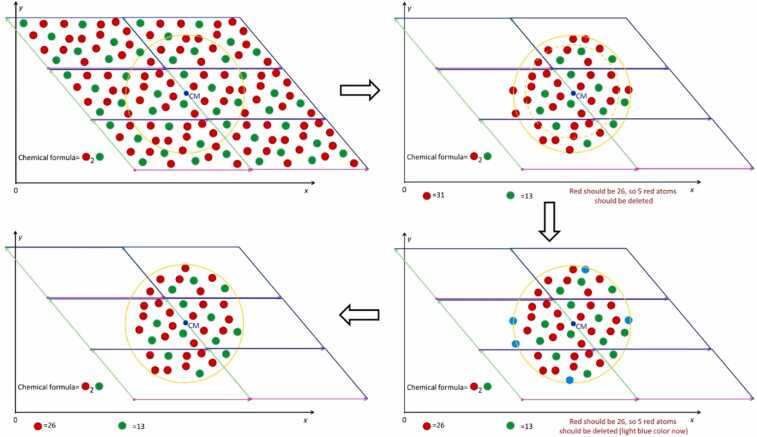
Graphical illustration of the NP construction algorithm for an imaginary material consisting of red and green atoms with stoichiometry 2:1. The Parallelograms of this Figure show the boundaries of each unit cell which is replicated in space to make a larger parallelogram. Only the atoms inside the diameter of our NP are kept to construct our NP. If the stoichiometry of the atoms inside the sphere is different from the stoichiometry of the unit cell, the atoms that are in abundance are deleted to get the structure we will use for the next step of energy minimization. To find the outer atoms which are candidates to be deleted in order to achieve the desired stoichiometry (indicated as blue spheres in the bottom right step), an outer shell of 0.02 Å thickness was used.

Despite optimizing the replication of the unit cell in space to create an appropriate box, additional steps are required to digitally construct a spherical NP. The first step involves removing atoms from the box that lie outside the sphere's diameter. However, this method alone does not guarantee that the remaining atoms will maintain the stoichiometry necessary for a neutral and realistic NP. To address this, ASCOT identifies the species with a greater proportion of atoms relative to the total number indicated by its chemical formula. It then calculates the exact number of atoms to remove from each species to maintain the correct stoichiometry. Next, ASCOT locates the atoms within an inner sphere, which has a radius 0.02 Å smaller than the desired NP. Atoms in the box that fall within a shell of thickness 0.02 Å (i.e., outside the inner sphere) and belonging to the excess species are removed until the stoichiometry aligns with the chemical formula. If the number of excess atoms in the shell exceeds those needed for stoichiometry, random numbers with a specific seed are used to determine which atoms to remove ensuring consistent results for identical input parameters. Conversely, if the excess atoms are fewer, the shell's thickness is increased by a further 0.02 Å, and the process is repeated until stoichiometry is achieved. This procedure is detailed in Fig. 3 for a representative imaginary material. The chosen shell thickness of 0.02 Å ensures that outer atoms are removed without disrupting the NP's internal structure by creating unrealistic defects (e.g., removing an oxygen atom from the interior of a TiO_2_ sphere would incorrectly alter the titanium atom's coordination). Given that chemical bonds are approximately 1 Å or longer, a shell thickness of 0.02 Å is a conservative choice for the algorithm. However, this increases computational time, as a larger shell thickness expedites convergence to stoichiometry but compromises the realistic digital construction of the NP (i.e., the presence of unrealistic internal defects such as converting an octahedron to a bipyramid/tetrahedron that doesn't exist in the material's unit cell). If a user wishes to create a charged NP, they can start with the neutral NP constructed by ASCOT and manually remove outer atoms. This is feasible given that the surface charge density of NPs is typically less than 4 e/nm^2^ [34], and certain regions of a material preferentially have excess atoms [35]. Note that ASCOT does not allow the creation of spherical NPs with a radius smaller than the minimum edge of the unit cell because: a) this could lead to stoichiometrically inaccurate and non-neutral NPs, and b) the resulting spherical NP would heavily depend on the chosen centre position, adding further complexity for non-expert users.

**Fig. 4:**
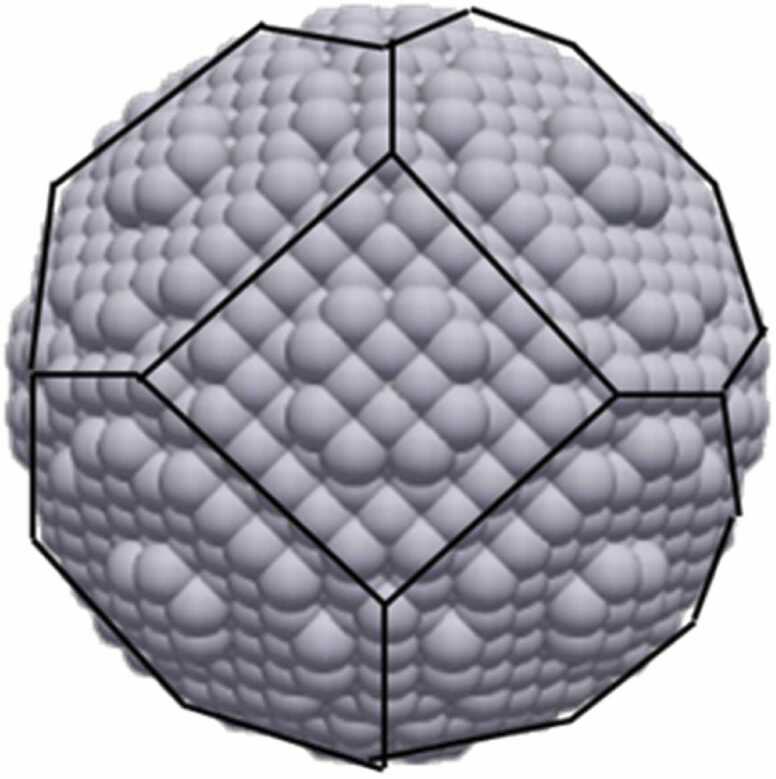
Geometrically constructed Native Silver NP created by ASCOT after inserting 5 nm diameter as input.

The geometric construction process as previously described above might result in a polyhedron rather than a sphere, with the input sphere circumscribing the polyhedron (as shown in Fig. 4). This outcome occurs because crystal atoms are not uniformly distributed in space and adhere to specific positional rules. Additionally, it's possible that the resultant NP is trapped in a local energy minimum. To achieve the global energy maximum, overcoming energy barriers is necessary. Atomistic Molecular Dynamics simulations at high temperatures (e.g., near the melting point) can facilitate this process [36], but due to their time-intensive nature, they cannot be integrated into a web application, which by its nature is constrained by time and computational resources. To enable users to investigate the global minimum structure, ASCOT provides the output in the form of LAMMPS datafiles for the constructed NPs. These datafiles can be fed into LAMMPS [21] for high-temperature molecular dynamics simulations. Moreover, a NP may behave differently in a solvent compared to a vacuum. The integration of a large number of solvent molecules around a NP in ASCOT is not feasible due to computational resource limitations. However, users can utilize ASCOT’s datafiles by adding solvent atoms themselves to investigate the solvent's effect on the NP structure and morphology. Special attention should be given to NPs in water due to their potential reactions (such as hydroxyl and hydrogen incorporation). This need for additional developments to enable integration of solvent will be included in an updated version of ASCOT.

### 2.3 Digital construction of energy minimized spherical Ag, CuO and TiO_2_ NPs

The geometrically constructed spherical NPs created by ASCOT, based solely on geometric considerations, may differ from their actual structures. This difference arises because they are constructed based on the bulk phase structure, which lacks outer atoms. The outer atoms of a NP experience a different chemical environment (e.g., varying numbers of neighbours, coordination numbers, potential energy and contact with solvent (although that is not explicitly considered in ASCOT as yet, as discussed in Section 2.2 above)) compared to the atoms in the bulk phase. However, the inner atoms of larger NPs are expected to resemble the bulk phase structure closely. These differing surface properties compared to the bulk phase make NPs suitable for various applications involving sensing and binding, including as catalysts. To construct a realistic spherical NP structure, energy minimization must be applied to the geometrically constructed NP. For energy minimization, a Force-Field, the functional form and parameter sets used to calculate the potential energy of a system of the atomistic level**,** is required. ASCOT utilizes the OpenKIM database of Interatomic Models [22] and provides a list of preselected Force-Fields tested for their ability to produce successful interaction descriptors for Ag, CuO, and TiO_2_ NPs. Not all OpenKIM Force-Fields are compatible with ASCOT’s code. ASCOT has screened the OpenKIM Force-Fields for these materials, ranking them from less to more generic based on chemical element relevance. The less generic a Force-Field is, the more specific it is to the NP’s chemical elements. This ranking aids non-experts in computational chemistry in selecting appropriate Force-Fields within ASCOT. The default choice in ASCOT is the least generic Force-Field. This setup allows users to create reliable NPs of their chosen composition and to obtain accurate results by merely selecting the material type and NP diameter. More experienced users can also choose a Force-Field from the tested list. The Force-Field name’s suffix indicates its OpenKIM database ID, while the prefix provides information about the Force-Field type. ASCOT employs various Force-Fields, including the Embedded Atom Model (EAM) [37], [38], the Modified Embedded Atom Model (MEAM) [39], the Buckingham potential [40], the Morse potential [41], an Effective Medium Theory potential, and the Charged Optimized Many-Body 3rd Generation (COMB3) Force-Field [42]. Each Force-Field type uses a different mathematical function. Notably, the COMB3 Force-Field, the default choice for TiO_2_ and CuO materials, is not sourced from OpenKIM but is Internally integrated into ASCOT. Since no OpenKIM Force-Fields for CuO were stable during minimization, the Liang et al. [42] Force-Field, covering elements like O, Cu, N, C, H, Ti, Zn, and Zr, was added to the list despite not being in OpenKIM. COMB3, a bond order potential, is preferred for covalently bonded solids, unlike EAM/MEAM, which are better suited for metals. COMB3 also assigns partial charges according to each atom's chemical environment using the Charge Equilibration method [43], although atomic polarization is ignored to save computational time. The presence of multiple Force-Field options in ASCOT is due to each being optimized for predicting specific properties of particular materials. All the Force-Fields in ASCOT are reactive, allowing bond breaking, crucial during energy minimization as surface atoms can form new bonds after losing outer neighbours in the geometric construction process. In addition to Force-Field selection, special treatment of the simulation box is necessary before applying energy minimization.

**Fig. 5:**
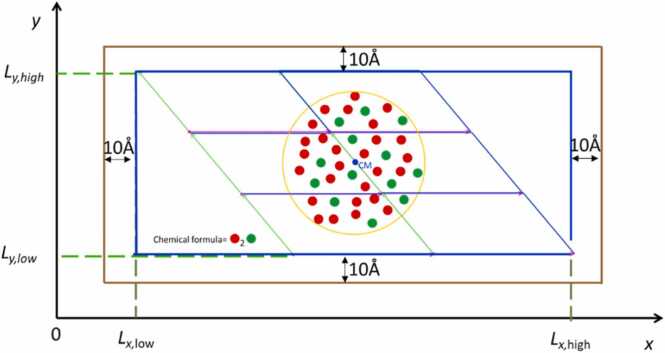
Conversion of a triclinic box to orthorhombic one and increase on its edge 10 Å to avoid NP self-interactions.

After the removal of excess atoms (as shown in Fig. 3 above), the simulation box in ASCOT is converted to an orthorhombic shape (as shown by the blue line in Fig. 5), and each edge is then extended by 10 Å. This rectangular box resulting from this extension (shown as the brown box in Fig. 5) is the cutoff value for force and energy calculations in ASCOT. Since ASCOT aims to construct a spherical NP in infinite dilution, it is crucial to exclude any self-interaction of the NP with its periodic images. By increasing each dimension of the box by 10 Å and maintaining the periodic boundary conditions, we ensure no interaction between the NP and its periodic images. This approach, by extending the edge length beyond the cutoff and considering only interactions within this range, allows ASCOT to simulate and minimize a NP as if it was in infinite dilution. Even when a long-range interaction Force-Field is selected, ASCOT employs a cutoff for all calculations (see Section 2.4) to prevent self-interactions of the NP and to save significant computational time and resources by avoiding lengthy long-range calculations. This optimization enables ASCOT to deliver results within a 10-second timeframe, which is essential for web tool functionality (e.g., to prevent loss of web connection). To maintain this efficiency, a limit of 10 nm has been set for the NP’s diameter currently, although access to larger spherical NPs is available upon request. Periodic boundary conditions are utilized to accommodate extreme cases, such as atoms preferring non-spherical shapes post-minimization (e.g., forming a periodic strip or expanding into a periodic bulk phase) and atoms expanding beyond the box limits. The conjugate gradient method is applied for energy minimization, halting when any of the following criteria are met: minimum energy tolerance (the unitless ratio of the energy difference between two consecutive steps to the energy value of the first step), minimum Force tolerance (the 2-norm length of the global 3*N*-dimensional Force vector consisting of the individual Force vectors of the *N* atoms of the NP), the maximum number of iterations, or the maximum number of Force evaluations.

### 2.4 Defining and calculating the constructed NP’s atomistic descriptors

NPs have distinct properties in their core and shell regions. Atoms residing within the inner part of the NP, or the "core," generally exhibit properties similar to those of the bulk material. In contrast, atoms in the outer layer or "shell" of the NP behave differently due to having fewer neighbours compared to core atoms. This difference in neighbour count also leads to structural variations between shell and core atoms, as shell atoms shift from their initial positions to achieve an energy-minimized structure [36]. Therefore, the geometrically constructed structure may differ significantly from the energy-minimized "real-world" structure, making a mere geometrical digital construction insufficient for calculating atomistic descriptors. An energy minimization procedure is thus necessary before calculating these descriptors, performed as described in 2, 3 above.

**Fig. 6:**
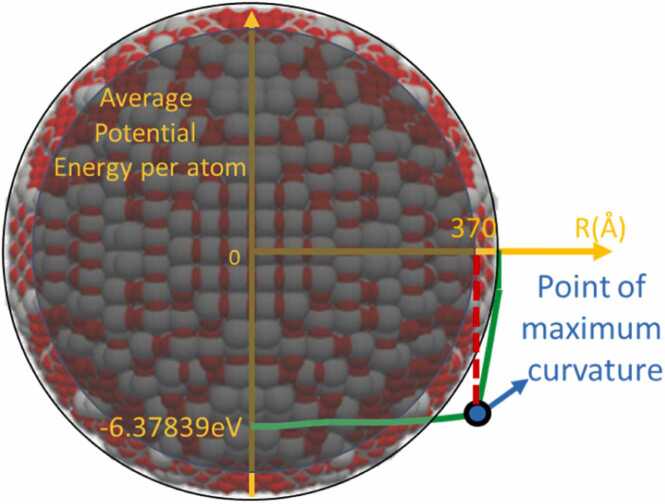
A TiO_2_ (Anatase) NP made by ASCOT having its diameter equal to 7.4 nm and a sketch of the Shell Average Potential Energy per atom as a function of the radius beyond which shell starts (see Burk et al. [44]). The blue point is the point of the maximum curvature of the Shell Average Potential Energy per atom which Burk et al. [44] used to determine the borderline between the core (dark sphere) and the shell (the region between the outer and the inner sphere) according to the Kneedle algorithm [46].

Following the energy minimization process, the average potential energy per atom and the average coordination number per atom can be calculated as a function of their distance from the centre of the NP, as shown in Fig. 6. These calculations are used to define the shell and core regions of the NP, following the approach of Burk et al. [44] and Tämm et al. [45], and their Shell Depth Calculator (https://nanogen.me/shell-depth), which is specifically applied to metal oxides (i.e., TiO_2_ and CuO in this case). According to this method, the point of maximum curvature in these properties can define the two regions using the Kneedle algorithm [46], predicting shell depths of 4 Å for TiO_2_ (Anatase), 4 Å for CuO, and 5 Å for TiO_2_ (Rutile). For these calculations, ASCOT constructed NPs with diameters of 3 and 4 nm for TiO_2_ (Anatase), 5 and 6 nm for CuO, and 3 and 5 nm for TiO_2_ (Rutile). The resulting XYZ files from ASCOT were then used as input for the Shell Depth Calculator. Given that the shell depths of the ASCOT materials are similar, a constant shell depth value of 4 Å was adopted for every material in ASCOT to avoid bias in the descriptor values due to varying shell depths. Although the overall NP maintains the material's stoichiometry, the stoichiometry of the core and shell regions may differ from that of the bulk material.

With the NP's atoms classified into "shell" and "core" types, atomistic descriptors for each type, as well as for the entire NP, can be calculated. These descriptors include: a) the logarithm of the number of atoms, b) average potential energy per atom, c) average coordination number, d) NP diameter, e) surface area, f) volume, g) lattice energy, h) average common neighbourhood parameter (CNP) as defined by Tsuzuki et al. [47], i) average first and second hexatic order parameters as defined by Nelson and Halperin [48]. Additionally, the ratio and/or difference between the core and shell descriptors are indicative of the NP's properties, signifying the extent of surface variation from the core. For instance, a significant difference in coordination number and/or potential energy between core and shell atoms suggests a highly reactive surface. While these specific descriptors are not included in ASCOT, they can be manually calculated using the descriptors calculated by ASCOT.

For the calculation of the average coordination number and the common neighbourhood parameter (CNP) descriptor in ASCOT, a cutoff value is necessary to distinguish between first neighbours and others. For Ag (native silver), where only one chemical element is present, this value is set at 2.4 times the Ag ionic radius. In cases where two different elements are involved, such as TiO_2_ and CuO, the cutoff is 1.2 times the sum of their ionic radii, with ionic radii defined as proposed by Shannon [49]. This approach includes atoms contributing to chemical bonds within the coordination number, like the 12 bonds in native silver. However, for metal oxides with corner-edged octahedra, coordination includes nearby non-bonded atoms. For example, in TiO_2_ Rutile (refer to Fig. 1(d)), a Ti atom is bonded to six oxygen atoms (Ti coordination number equals to 6), an oxygen atom is bonded with three Ti atoms and it is also close to another O atom (where O’s coordination number equals to 4) resulting in an average coordination number of 4.66, considering the double count of Ti atoms by oxygen due to the chemical formula (TiO_2_).

Furthermore, the CNP is a valuable metric for assessing the local crystal structure around an atom, helping to determine whether an atom forms part of a perfect lattice or is a local defect. According to CNP formula [47], a value near zero indicates a highly symmetrical structure, typical of materials like Face-Centre Cubic (FCC) (typical for native Ag) and Body-Center Cubic (BCC) and those with high-symmetry space groups.

The first and second hexatic order parameters, representing the real and imaginary parts of a hexatic order metric as proposed by Nelson and Halperin [48], measure hexagonal symmetry. These parameters can be used to derive other descriptors, such as the phase and magnitude of the polar representation of this complex number metric. An absolute magnitude significantly smaller than 1 suggests weak hexagonal symmetry of the neighbours of an atom around it.

The potential energy per atom is calculated as the sum of each atom's contribution to various pair, bond, and other potentials in the selected Force-Field. Each atom's contribution is assumed to be half for pair and bond interactions, one-third for angle interactions, and one-fourth for dihedral interactions. This definition ensures that the sum of the potential energy per atom across all atoms equals the system's total potential energy. The Electric Quadrupole [50], which could describe the interaction of a neutral NP with an electric field, is not included in ASCOT due to the requirement for partial charges and the non-utilization of such forces in some Force-Fields like EAM and MEAM. Additional descriptors can be calculated from existing ones by users. For instance, the first and second derivatives of ASCOT descriptors, obtained through finite difference formulas for NPs of nearby diameters, can indicate the favorability of crystal growth and the reactivity of the NP's surface. The user guide contains suggestions for additional parameters that can be calculated.

## 3 Description of ASCOT’s Graphical User Interface and its integration into the Enalos Cloud Platform [51]

**Fig. 7:**
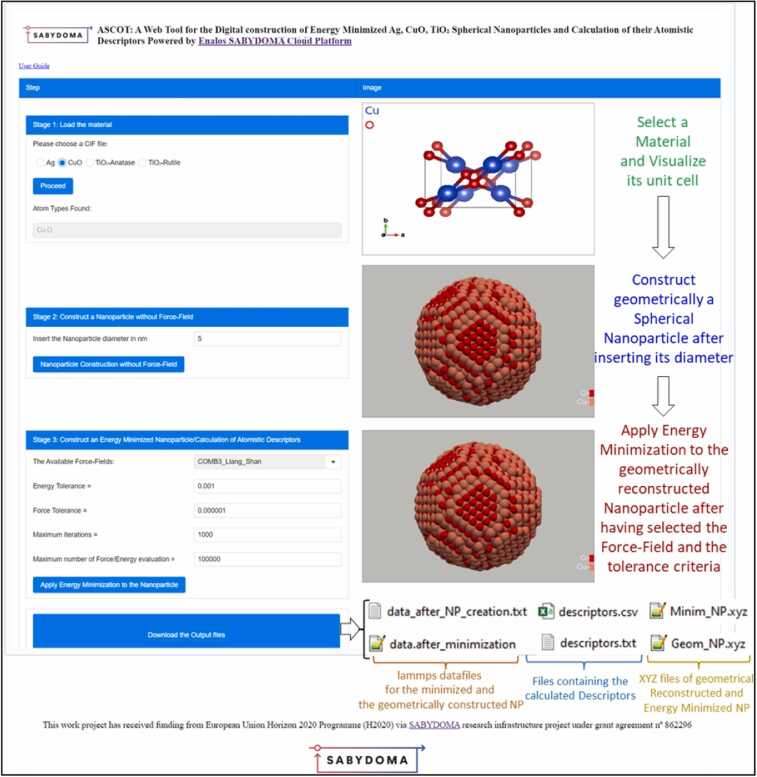
Graphical User Interface of ASCOT with a description of the NP generation and optimization Stages and its derived files, demonstrated as implementation of the construction of spherical CuO NPs having diameter equal to 5 nm, and the energy minimisation step. The descriptors are then automatically calculated for the energy minimised NPs.

ASCOT combines the aforementioned tools and methodology for construction of *in silico* NPs and calculation of their descriptors with a Graphical User Interface (GUI) as shown in Fig. 7, enabling non-expert users to digitally construct Ag, CuO, and TiO_2_ (anatase, rutile) NPs and calculate their atomistic descriptors. Users can easily select the NP composition (material), diameter, and a Force-Field from a pre-selected drop-down list, without needing in-depth knowledge of the background calculations. As depicted in Fig. 7, ASCOT's process is divided into three stages. In the first stage, upon selecting the material composition of interest and clicking the proceed button, the unit cell of the material automatically appears on the right side of the screen. In the second stage, the user inputs the required NP diameter, triggering backend algorithms (as described in Fig. 2, Fig. 3) to digitally and geometrically construct the spherical NP. Once this digital construction is complete, an image of the geometrically constructed NP is displayed on the user’s screen. The xyz file containing the NP coordinates can be downloaded by clicking the “Download the Output Files” button at the bottom of the webpage. The resulting output file contains the original CIF file and its metadata as well as a unique ID for the constructed NP and its Force-Field and energy minimisation steps, and the x,y,z information for each atom in the particle.

However, as previously mentioned, the structure of geometrically constructed NPs may differ from their actual structures, particularly at the surface. Therefore, in the third and final stage of ASCOT, energy minimization is applied to the NPs. Users can scroll through and select from different Force-Fields in the GUI, as well as alter or accept the default stopping criteria for energy minimization. This flexibility allows users to explore the impact of various Force-Fields on the descriptors and on any subsequent Machine Learning model applied to the in silico NPs and/or their descriptors. After selecting a Force-Field and setting the tolerance criteria, clicking on the “Apply Energy Minimization to the Nanoparticle” button will initiate the process, and the energy-minimized NP will be displayed on the right side of the screen (as shown in Fig. 7). Subsequently, users can download a ZIP file containing all the files generated by ASCOT, including LAMMPS datafiles, XYZ files of both geometrically and energy-minimized constructed NPs, the images of the geometry and energy minimised NPs and the descriptor csv and txt files.

ASCOT is hosted by the Enalos Cloud Platform to achieve its synergistic combination with the rest of the tools available on the platform (e.g., the comprehensive suite of predictive models, which are adeptly provided as web services). The Enalos Cloud Platform, developed by NovaMechanics Ltd, is an online resource in the area of cheminformatics/nanoinformatics, and notably, it is freely accessible to the scientific community through user-friendly GUIs, embodying a significant advancement in open and democratised cloud-based scientific computation. This arrangement (which will be maintained for at least the next 5 years or longer if the technology has not shifted in a way that renders this untenable), coupled with the platform's powerful cloud computing capabilities, substantially reduces the barriers typically associated with complex scientific computations, including removing the requirement that users are familiar with computer programming languages. This is invaluable in facilitating advanced data analysis and modelling by a much wider range of users, thereby extending its utility to a broader range of scientific pursuits including SSbD and regulation.

A key attribute of the Enalos Cloud Platform is its ability to assimilate and integrate disparate data sources. This functionality is particularly advantageous in fields such as computer-aided drug discovery, materials design, and decision-making processes. The user-friendly environment of the platform is specifically designed to cater to non-informatics experts, granting them access to state-of-the-art modelling tools essential for hazard prediction and risk assessment in various scientific and industrial applications. Additionally, the platform's deployment of the Software as a Service (SaaS) model ensures that any computational model, including ASCOT, is not only more accessible but also user-friendly. This approach is particularly aligned with the current and evolving needs of researchers and scientists who require efficient, reliable, and easily navigable tools to advance their research. Consequently, the Enalos Cloud Platform, through its SABYDOMA ASCOT instance, represents a significant leap forward in scientific research and computation, offering a synergistic combination of advanced computational resources with the accessibility and convenience of cloud technology.

## 4 Implementation of ASCOT to calculate Ag, CuO and TiO_2_ NP’s atomistic descriptors and their atomic coordinates

**Fig. 8:**
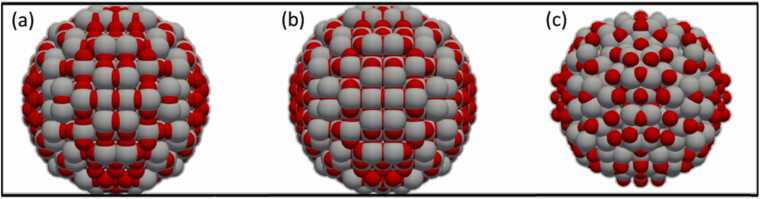
(a) Geometrically constructed spherical TiO_2_-Anatase NP of 3.5 nm diameter, (b) Energy-minimized spherical TiO_2_-Anatase NP of 3.5 nm initial diameter using a COMB3 type Force-Field [42] and (c) Energy-minimized spherical TiO_2_-Anatase NP of 3.5 nm initial diameter using a MEAM type Force-Field of Zhang and Trinkle [52].

The influence of energy minimization on geometrically constructed Anatase TiO_2_ NPs of 3.5 nm diameter has been analysed using two different Force-Fields [42], [52]. A comparison of the geometrically constructed and energy-minimized Anatase TiO_2_ NP's surface using the COMB3 Force-Field [42], as depicted in Fig. 8, reveals that the COMB3 Force-Field slightly alters the position of surface oxygen atoms, moving them inward to minimize the NP's energy. While COMB3 induces only minor changes, the MEAM type Force-Field developed by Zhang and Trinkle [52] significantly alters the NP's surface and causes the NP to expand by 4.5% in *x* and y direction and by 2.7% in z direction compared to the diameter of the geometrical constructed NP, as seen in Fig. 8. This variation indicates that selecting an appropriate Force-Field is crucial for realistically constructing NPs, even if both of the Force-Fields are optimized for TiO_2_ properties. The differences in Anatase TiO_2_ NP surfaces between these two Force-Fields might be due to a local minimum in the COMB3 [42] Force-Field or a failure of the MEAM type Force-Field [52] to realistically describe the NP surface. Overcoming a local minimum would require molecular dynamics simulations at higher temperatures [36], but such an extensive investigation falls outside ASCOT's scope. Thus, for less expert users, ASCOT recommends the default Force-Field, COMB3.

**Fig. 9:**
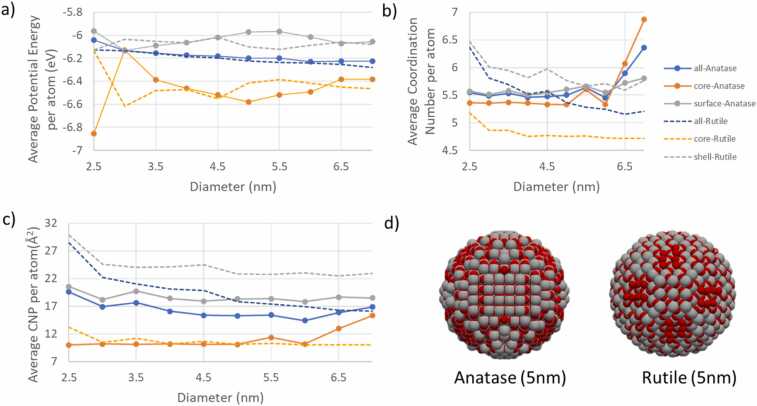
The average potential energy (a), the average coordination number (b) and the average CNP number (c) per atom as a function of the diameter of Rutile and Anatase NPs. Solid and dashed lines illustrate the descriptor values for the Anatase and the Rutile phases of TiO_2_ NPs respectively. The whole NP, the core of the NP and the shell of NP descriptors are illustrated with blue, orange, and grey line colours respectively in (a-c). Anatase and Rutile NPs having diameter equal to 5 nm are also illustrated (d), with the Ti and O illustrated with grey and red coloured balls, respectively.

In addition to constructing the various NPs, ASCOT has been employed to calculate the range of descriptors outlined in Section 2.4. A series of NPs (Ag, CuO, TiO_2_-Anatase, and TiO_2_-Rutile) with diameters ranging from 2.5 - 7.0 nm were investigated. For TiO_2_ NPs (both rutile and anatase), descriptors such as: a) average potential energy per atom, b) average coordination number per atom, and c) average CNP per atom were calculated, with their values illustrated in Fig. 9 as a function of the NP diameter. The average potential energy per atom decreases with increasing NP size, suggesting favourable crystal growth for both Anatase and Rutile up to 7 nm diameter. As shown in Fig. 9, the average potential energy of core atoms ranges between − 6.6 and − 6.4 eV per atom, slightly higher than the value of − 7.2 eV reported by Yang et al. [53], corresponding to a cohesive energy per TiO_2_ bulk anatase of − 21.60 eV, which can be calculated using Eq. (1):

(1) E_coh_(TiO_2_) = E_tot_(TiO_2_)- E(Ti) −2E(O)

The cohesive energy is an important parameter that describes the strength of metal bonds in NPs, which is equal to the energy of splitting the NPs back into individual atoms [54].

**Fig. 10:**
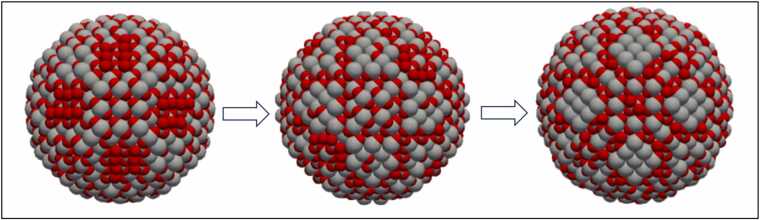
Crystal growth of TiO_2_-Rutile NP starting from a diameter of 5 nm (left), growing into a NP having diameter equal to 5.2 nm (middle) and 5.4 nm (right). Ti and O are illustrated with grey and red colours, respectively.

Fig. 9 suggests that the Rutile TiO_2_ NPs are slightly more stable than the Anatase TiO_2_ NPs, aligning with the known stability of bulk rutile phase in a vacuum according to Hanaor and Sorell [30]. Incrementing the diameter in smaller steps allows observation of crystal growth, as illustrated in Fig. 10. This figure also indicates that a slight diameter increase (about 0.2 nm) leads to different surface structures in the NP. Despite similar average potential energy profiles for Rutile and Anatase as per Fig. 9, the average coordination number and CNP per atom differ for these two phases. The CNP values for Anatase converge near 7 nm, showing that NP size significantly influences this descriptor. Using descriptors is a method to measure structural/optical differences between Anatase and Rutile NPs, as shown in Fig. 9-d. A nonzero CNP number indicates a lack of symmetry in the coordinated atoms, with larger CNP numbers on the surface than in the core, reflecting the absence of outer atoms on the surface not counteracting the inner coordinated atoms' contribution.

**Fig. 11:**
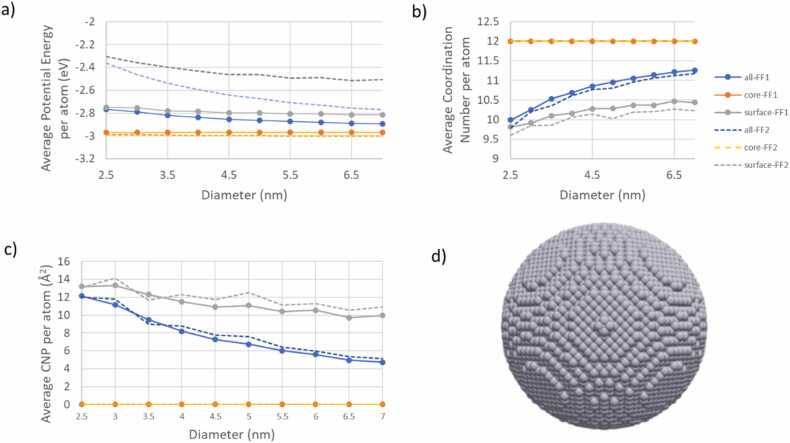
The average potential energy (a), the average coordination number (b) and the average CNP number (c) per atom as a function of the diameter of Ag NPs. Solid and dashed lines illustrate the descriptors values for the Force-Fields of Ackland et al. [55] and Girifalco and Weizer [56]*,* respectively. The whole NP, the core of the NP and the shell of NP descriptors are illustrated with blue, orange, and grey colours respectively in (a-c). An Ag NP having diameter equal to 9 nm is also illustrated (d).

In the study of Ag NPs, the descriptors obtained using the Force-Fields of Ackland et al. [55] and Girifalco and Weizer [56] were examined to assess the impact of the different Force-Fields. Both Force-Fields yield an average potential energy per core atom near 2.95 eV, aligning with the experimental cohesive energy value for Ag reported in the literature [57], [58]. However, the average potential energy per surface atom shows a variance of about 0.3 eV between these Force-Fields across the range of diameters investigated. Despite this difference, it does not seem to result in a distinct structure change for the silver NPs, as both the average coordination number and the average CNP number per atom are closely aligned according to Fig. 11 b) and c). As the diameter of the Ag NP increases, the coordination number approaches the value for its core, which is also indicative of its bulk phase. This trend suggests decreased reactivity for larger NPs due to fewer active (uncoordinated) sites, a pattern that was less pronounced in the TiO_2_ NPs (Fig. 9). Unlike the fluctuating descriptor values of the TiO_2_ core atoms, the descriptors for Ag core atoms remain consistent with increasing NP diameter, mirroring the properties of their bulk phase. This consistency strongly suggests that the core atoms of Ag have the same structure as their bulk phase atoms, even in very small (2.5 nm) Ag NPs.

**Fig. 12:**
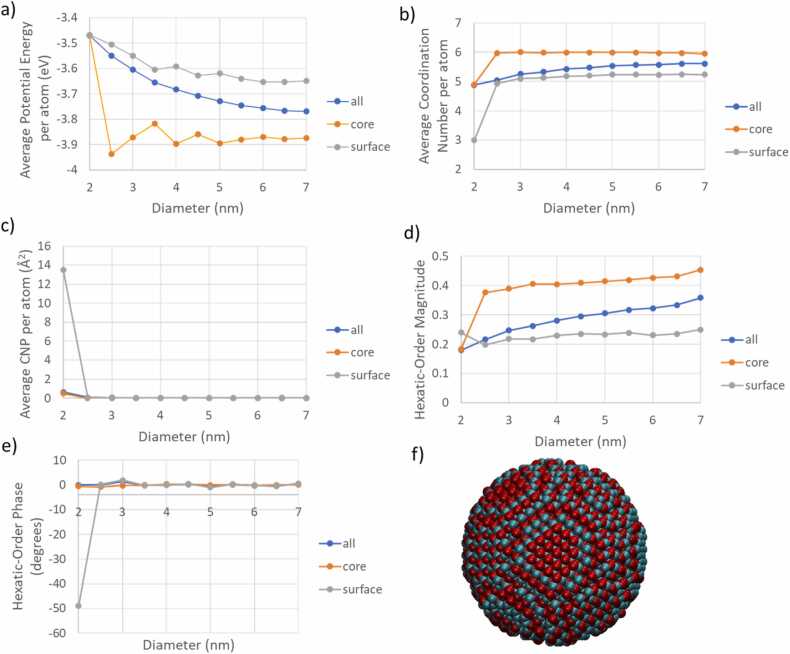
The average potential energy (a), the average coordination number (b) the average CNP number (c) per atom as a function of the diameter of CuO NPs for the COMB3 Force-Field [42], the magnitude of the hexatic order parameter (d) and its phase (e). The whole NP, the core of the NP and the shell of NP descriptors are illustrated with blue, orange, and grey colours, respectively, in (a-e). A CuO NP geometrically constructed having diameter equal to 5 nm is also illustrated (f) with the Cu and O shown as green and red respectively).

Regarding CuO (tenorite) NPs, the descriptors appear to plateau beyond a diameter of 3 nm as shown in Fig. 12, indicating that their properties and reactivity do not significantly change with increasing NP size, thus implying consistent reactivity. The average potential energy per atom suggests that crystal growth is favourable up to a diameter of 7 nm. The near-zero CNP number points to symmetry in the coordination of its atoms, not just in the core but also on the surface. This symmetry is reminiscent of hexagonal symmetry, as inferred from the magnitude of hexatic order in Fig. 12. Additionally, the zero phase of hexatic order suggests that this hexagonal symmetry extends along the x-direction of the unit cell.

## 5 Discussion

The ASCOT web tool addresses the need for further systematic investigation into the toxicity of Ag, CuO, TiO_2_ (Anatase), and TiO_2_ (Rutile) NPs to enable design of safer and sustainable materials, by providing *in silico* NP specifications and their associated atomistic descriptors for use as inputs for development of predictive models of NP toxicity. Besides toxicity, ASCOT descriptors can be used for the prediction of other endpoints including biomolecule binding, and functional properties. Core atoms exhibit different descriptor values (average potential energy per atom, average coordination number, average CNP) than shell atoms and there is a potential to relate these differences to the toxicity of the NPs or to other endpoints (e.g., catalytic activity, sensing and binding of biomolecules etc.). Core atoms act behave like the atoms in the bulk phase of the material (i.e., have similar descriptors values) in contrast to the shell atoms that differ due to not having their valence shell complete.

Machine learning models and meta-models, developed using ASCOT-generated descriptors, could potentially eliminate the need for animal testing without compromising prediction quality, as the predictions are validated based on high quality, relevant and comparable historical experimental data. The large surface-to-volume ratios of NMs offers opportunities for enhanced interfacial interactions compared to traditional bulk materials. Through molecular modelling, ASCOT quantifies how this ratio influences NP properties and descriptors, based on atomistic Force-Fields. Deviations of the NP’s structure produced after applying different Force-Fields are attributed to: a) the different chemical environment that these Force-Fields may have been optimized in, and b) the trade-off between accuracy and computational time/computational resources. For smaller NPs, higher accuracy Force-Fields such as COMB3 [42] can be used, however, the use of COMB3 is prohibitive for NPs having diameter larger than 50 nm.

## 6 Conclusions

In this study, we introduced the ASCOT web tool, designed to simplify the construction of realistic spherical NPs of Ag, CuO, and TiO_2_ (anatase, rutile), while also automatically calculating atomistic descriptors crucial for assessing the toxicity of these NPs. We thoroughly analysed the ASCOT algorithm, elucidating the process of NP geometric construction, energy minimization, and the subsequent calculation of atomistic descriptors. We evaluated the atomistic descriptors of these four exemplar NPs across a range of sizes, beginning from a diameter of 2.5 nm and incrementally increasing by 0.5 nm up to 7.0 nm. Our focus was particularly on the average potential energy per atom descriptor to understand the crystal growth dynamics of these NPs. It was observed that crystal growth is favoured for all types of NPs, at least up to the maximum diameter of 7 nm investigated in this study. Notably, there was a strong correlation between the cohesive energies predicted by ASCOT and those obtained from experimental data, highlighting the accuracy and reliability of the ASCOT tool.

A critical aspect of our findings is the importance of Force-Field selection. The choice of Force-Field significantly affects the surface representation of the NPs and plays a crucial role in the accuracy of the calculated atomistic descriptors. Moreover, the division of the NP into two distinct regions - the core and the shell/surface - during descriptor calculation allows for a more detailed and nuanced understanding of the NP's structure and properties. This segmentation is vital for accurately characterizing the physical and chemical behaviour of the NPs, particularly in applications related to their toxicity and environmental impact. The ASCOT tool offers both ease of use for non-experts and robust, detailed analysis for researchers delving into the complexities of NP behaviour and characteristics.

Data generated by ASCOT is fully traceable (full provenance information provided as part of the downloadable data and accompanying metadata) and can be easily integrated into the ready for computation database NanoPharos [23] for further use in any of the Enalos tools for data analysis and computational prediction of nanomaterials properties, interactions and effects. The datasets generated as part of this paper, to demonstrate the power and utility of ASCOT are available via NanoPharos [23] through the link https://db.nanopharos.eu/Queries/Datasets.zul?datasetID= 17.

## Funding

This work has received funding from the European Union’s Horizon 2020 Research and Innovation Programme via the SABYDOMA project (grant agreement nº 862296) and from the European Union’s H2020 Marie Skłodowska-Curie Actions via CompSafeNano under (grant agreement nº 101008099).

## CRediT authorship contribution statement

Conceptualization: PK; Methodology: PK, EV Software: PK, NS, AT Supervision: GM, KT, IL, AA Writing – original draft: PK; Writing – review & editing: PK, EV, NS, AT, GM, KT, IL, AA Funding acquisition:, KT, IL, AA.

## Declaration of Competing Interest

PK, EV, NS, AT, AA are affiliated with NovaMechanics, a cheminformatics and materials informatics company.
